# An Essential Role for Calnexin in ER-Phagy and the Unfolded Protein Response

**DOI:** 10.3390/cells13171498

**Published:** 2024-09-06

**Authors:** Daniel Wolf, Chiara Röder, Michael Sendtner, Patrick Lüningschrör

**Affiliations:** Institute of Clinical Neurobiology, University Hospital Wuerzburg, Versbacher Str. 5, 97078 Würzburg, Germanychiara.roeder@stud-mail.uni-wuerzburg.de (C.R.)

**Keywords:** ER-phagy, unfolded protein response, UPR, calnexin, ER stress

## Abstract

ER-phagy is a specialized form of autophagy, defined by the lysosomal degradation of ER subdomains. ER-phagy has been implicated in relieving the ER from misfolded proteins during ER stress upon activation of the unfolded protein response (UPR). Here, we identified an essential role for the ER chaperone calnexin in regulating ER-phagy and the UPR in neurons. We showed that chemical induction of ER stress triggers ER-phagy in the somata and axons of primary cultured motoneurons. Under basal conditions, the depletion of calnexin leads to an enhanced ER-phagy in axons. However, upon ER stress induction, ER-phagy did not further increase in calnexin-deficient motoneurons. In addition to increased ER-phagy under basal conditions, we also detected an elevated proteasomal turnover of insoluble proteins, suggesting enhanced protein degradation by default. Surprisingly, we detected a diminished UPR in calnexin-deficient early cortical neurons under ER stress conditions. In summary, our data suggest a central role for calnexin in orchestrating both ER-phagy and the UPR to maintain protein homeostasis within the ER.

## 1. Introduction

In neurons, the endoplasmic reticulum (ER) forms a highly dynamic network that enters axons and presynaptic terminals and plays a central role in protein translation, Ca^2+^ homeostasis, and synapse maintenance [1,2,3,4,5]. ER stress is a major pathophysiological pathway linked to neurodegenerative disorders such as amyotrophic lateral sclerosis (ALS) [6]. ER stress triggers the unfolded protein response (UPR), a physiological signaling cascade that inhibits protein translation, induces the degradation of misfolded proteins, and facilitates protein folding within the ER [7].

In mammalian cells, the UPR consists of three pathways, initiated by ER transmembrane proteins that sense misfolded proteins: inositol-requiring enzyme 1 α (IRE1α), protein kinase RNA-like endoplasmic reticulum kinase (PERK), and activating transcription factor 6 (ATF6). All three sensors contain ER luminal domains that detect unfolded proteins and cytosolic domains which induce downstream signaling cascades to protect cells from ER stress. During its initial phase, the UPR is protective, but under prolonged ER stress conditions, the UPR promotes apoptosis [6,7]. Both IRE1α and PERK are type I transmembrane proteins with kinase activity, which oligomerize and autophosphorylate upon activation [8,9]. As an immediate adaptive response, PERK phosphorylates eukaryotic translation initiation factor 2 subunit α (eIF2α), triggering a transient attenuation of protein synthesis to prevent the influx of newly synthesized proteins into the ER [10,11]. Autophosphorylation of IRE1α initiates the activation of its RNase activity, resulting in the excision of a small 26-nucleotide intron from the mRNA encoding the transcription factor X-box-binding protein 1 (XBP1) [12,13]. Therefore, the translational open reading frame shifts, which results in the expression of an active XBP1 transcription factor (XBP1s) driving the expression of genes involved in ER protein translocation, folding, and secretion, as well as the degradation of misfolded proteins [7,14]. In contrast to IRE1α and PERK, ATF6 is a type II transmembrane protein. Full-length ATF6 (ATF6p90) transits from the ER to the Golgi apparatus in response to ER stress. At the Golgi apparatus, it is cleaved by site-1 and site-2 proteases to generate a fragment containing a basic leucine zipper transcription factor (ATF6p50), which translocates to the nucleus to induce gene expression [15,16,17].

The ER-resident protein calnexin is a lectin transmembrane type I protein. Besides its membrane-spanning region, calnexin possesses a glycan-binding domain, the P-domain, and a C-terminal cytosolic region [18]. The lectin domain recognizes and binds specific glycosylation patterns of nascent glycoproteins which represents the first step of the calnexin/calreticulin cycle. Calreticulin represents the homologous counterpart of calnexin within the lumen of the ER [19,20]. During the calnexin/calreticulin cycle, newly synthesized glycoproteins bind to calnexin and calreticulin for proper folding within the ER [21,22]. Besides its function as an ER chaperone, calnexin is involved in several non-canonical functions [23,24].

Recent work suggested a central role for calnexin in directing misfolded proteins to ER-to-lysosome-associated degradation (ERLAD) by ER-phagy [25,26,27], a specialized form of autophagy, defined by the constitutive or regulated clearance of ER portions by lysosomal degradation [28]. Calnexin recognizes misfolded endogenous procollagen and delivers it to ER-phagy by interaction with the ER-phagy receptor FAM134B [26]. Calnexin also assists in the removal of misfolded proteins in the ER which are resistant to proteasomal degradation via ER-associated degradation (ERAD) [25]. As shown for antitrypsin Z (ATZ) and procollagen, the persistent association with calnexin and continuous cycles of de-/re-glycosylation are required and sufficient to label for FAM134B-mediated ERLAD. Furthermore, calnexin acts as a switch for targeting the neurotrophin receptor TrkB either to the cell surface or towards autophagosomal processing. In cortical precursor cells, depletion of calnexin leads to impaired cell surface transport of TrkB and targeting from the ER to lysosomal compartments via the ER-phagy receptor FAM134B [24]. Taken together, calnexin appears as a multi-functional ER chaperone, which can integrate cytosolic signals with ER homeostasis.

In neurons, the ER extends into dendrites and axons [1,2,3,4,5]. The function of such axonal ER has been suggested to be limited to lipid metabolism, Ca^2+^ homeostasis, and functions in contacting membranous organelles to regulate their biogenesis and maintenance [2,4,5]. Traditionally, axons were considered to exhibit only smooth ER, while lacking rough ER [29]. Therefore, the role of the axonal ER in local protein synthesis and folding is less characterized. However, in response to extracellular stimuli, ribosomes assemble into 80S subunits within axon terminals and associate with the ER within seconds, suggesting a highly dynamic function of the ER in local protein translation [1]. Interestingly, this highly dynamic interplay between ribosomes and the ER in axon terminals of cultured motoneurons contributes to impaired local protein synthesis of spinal muscular atrophy [3]. In summary, these studies point to a critical role of the ER in local protein translation, raising the question of local mechanisms such as the UPR and ER-phagy to counteract axonal ER stress.

Here, we will show that the chemical induction of ER stress triggers ER-phagy in the somata and axons of cultured motoneurons (MNs). Under basal conditions, the depletion of calnexin leads to enhanced ER-phagy in the axons of cultured MNs. However, calnexin-deficient cells showed impaired ER-phagy upon ER stress induction. In addition to increased ER-phagy, we also detected an elevated proteasomal turnover of insoluble proteins, suggesting enhanced protein degradation. Surprisingly, we observed a diminished UPR in calnexin-deficient early cortical neurons upon ER stress induction. In summary, our data suggest a central role for calnexin in orchestrating ER-phagy and the UPR to maintain protein homeostasis within the ER.

## 2. Materials and Methods

### 2.1. Animals

Calnexin knockout (*canx^−/−^*) mice [30] were bred on a C57BL/6J background and housed either at 4–6 animals per cage or in pairs for breeding. Animals were kept under a 12 h/12 h light/dark cycle at a room temperature of 20–22 °C and 55–65% humidity with unrestricted access to food and water. The mice were genotyped using PCR analysis. All experiments were performed following animal protection regulations according to the German federal law under the supervision of the local veterinary authority (Veterinaeramt Stadt Wuerzburg) and the Committee on the Ethics of Animal Experiments, i.e., Regierung von Unterfranken, Wuerzburg, Germany.

### 2.2. Cortical Precursor Cell Culture

The culture of cortical precursor cells was performed as previously described [24]. In brief, tissue of the prefrontal cortex was dissected from (*canx^−/−^*) mice at the developmental stage E13 and the tissue was digested using 0.1% trypsin (#LS003707; Worthington, Lakewood, NJ, USA) in 180 µL HBSS (14170088; Thermo Fisher Scientific, Waltham, MA, USA). After digestion, 0.1% trypsin inhibitor (T6522; Sigma-Aldrich, Munich, Germany) was added, and cells were separated using trituration. Cells were then transferred to 50 mL flasks (#690160; Greiner Bio-One, Frickenhausen, Germany) and cultured in 5 mL neurobasal medium (#21103-049; Thermo Fisher Scientific, Waltham, MA, USA) containing 20 ng/mL epidermal growth factor (EGF) (AF-100-15; PeproTech, Hamburg, Germany), 20 ng/mL fibroblast growth factor (FGF) (#100-18B; PeproTech, Hamburg, Germany), 1 × B27^TM^ (#A3582801; Thermo Fisher Scientific, Waltham, MA, USA), and 1:100 penicillin–streptomycin-glutamine (#15070063; Thermo Fisher Scientific, Waltham, MA, USA) at 37 °C and 5% CO_2_. After growth and passaging for 6 days, cells were transferred to 10 mm glass cover slips (#0111500; Paul Marienfeld, Lauda-Koenigshofen, Germany) for immunocytochemistry or 12-well plates (#83.3921; Sarstedt, Nuembrecht, Germany) or 6 cm dishes (#734-0961; VWR, Leicestershire, UK) for RNA or protein extraction. At a confluency of 70–80% and 12 h prior to experiments, cells were deprived of EGF and FGF. All glass cover slips and cell culture plates were coated with poly-DL-ornithine (PORN) (#P8638; Sigma-Aldrich, Munich, Germany).

### 2.3. Primary Motoneuron Culture

Primary MNs were cultured as previously described [31]. Briefly, the spinal cord of E12.5 embryos was dissected and incubated for 15 min in 0.1% trypsin in Hank’s balanced salt solution. The tissue was triturated and incubated in neurobasal medium (Invitrogen, Carlsbad, CA, USA), supplemented with 1 × Glutamax (Invitrogen, Carlsbad, CA, USA) on Nunclon plates (Nunc) pre-coated with antibodies against the p75 NGF receptor (MLR2; kind gift of Robert Rush, Flinders University, Adelaide, Australia) for 45 min. The plates were washed three times with neurobasal medium, and the remaining MNs were recovered with depolarization solution (0.8% NaCl, 35 mM KCl, and 2 mM CaCl_2_) and collected in MN medium (2% horse serum, 1 × B27 in neurobasal medium with 1 × Glutamax).

MNs were plated at a density of 20,000 cells per 10 mm cover slip. Directly before plating, the cells were transduced with the lentiviral vector FUV-mRFP-GFP-KDEL in 50 µL of MN medium.

### 2.4. Plasmid Construction

FUW-mRFP-GFP-KDEL was generated by the digestion of the FUW-TDP43-HA plasmid by BamHI and XbaI, and the resulting 10 kB fragment was purified by gel extraction. The cDNA of mRFP-GFP was amplified by PCR using FUW-mRFP-GFP-LC3 as the template with primer including the sequence encoding the KDEL signal peptide. The PCR product was subcloned into the pCMV-ECS2-CMV-myc-ER vector, which contains a secretory leader peptide for proper ER targeting, as previously described [1,32] The resulting PCR product was finally cloned into the FUW backbone by ligation.

### 2.5. Lentivirus Production

The plasmid constructs used for lentivirus production were generated as previously described [33]. Lentiviral particles were generated by co-transfecting HEK 293T cells simultaneously with the viral packaging plasmids pCMV-dR8.91 and pCMV-VSV-G and either the IRE1-3F6HGFP or ATF6-GFP plasmid, using calcium phosphate precipitation. After 24 h, the cell culture medium was replaced and 48 h later, the supernatant culture medium was used for virus concentration via ultracentrifugation.

### 2.6. Lentiviral Transduction for IRE1α and ATF6 Localization

For analysis of IRE1α and ATF6 localization, cells were transduced using the respective lentiviruses before plating 10,000 cells on PORN-coated glass cover slips. After 2.5 days, cells were EGF- and FGF-deprived and 12 h later were treated with either tunicamycin (#T7765; Sigma-Aldrich, Munich, Germany) at a final concentration of 1.5 µg/mL or dithiothreitol (DTT) (#20291; Thermo Fisher Scientific, Waltham, MA, USA) at a final concentration of 5 mM for the indicated time spans.

### 2.7. Immunocytochemistry (ICC)

Cells were fixed at the indicated time points after treatment with ER stress-inducing agents with 2% paraformaldehyde (wt/vol) (#104005; Merck, Darmstadt, Germany) in PBS for 15 min at room temperature (RT). Cells were washed twice with PBS, then once with TBS-T. Permeabilization and blocking was performed in one step using a blocking solution (10% donkey serum (vol/vol) (#C06SB; Bio-Rad, Munich, Germany); 0.3% Triton^TM^ X-100 (vol/vol) (#X100; Sigma-Aldrich, Munich, Germany); 0.2% Tween^®^ 20 (vol/vol) (#P1379; Sigma-Aldrich, Munich, Germany) in TBS-T) for 30 min at RT. Primary antibodies were added overnight at 4 °C in blocking solution. The antibodies used were the following: chicken anti-GFP (#ab13970, 1:1000; Abcam, Berlin, Germany), mouse anti-Tuj1 (1:1000; Neuromics, Edina, MN, USA), rat anti-Lamp1 (1:20; DSHB), and mouse anti-GM130 (#610822, 1:200; Becton Dickinson, Heidelberg, Germany). Then, cells were washed three times with TBS-T, and fluorophore-coupled secondary antibodies were added for 1 h at RT in TBS-T. The antibodies used were the following: donkey anti-chicken Alexa Fluor 488 (#703-545-155, 1:800; Jackson ImmunoResearch, West Grove, PA, USA) and donkey anti-mouse Cy3 (#13131357, 1:800; Thermo Fisher Scientific, Waltham, MA, USA). Subsequently, cell nuclei were stained with DAPI (#MBD0015; Sigma-Aldrich, Munich, Germany) for 5 min. Cells were washed three times with H_2_O and mounted on glass slides with FluorSave^TM^ (#345789; Merck, Darmstadt, Germany).

### 2.8. Image Acquisition, Processing, and Analysis

#### 2.8.1. Acquisition

Images of ICC experiments were obtained using an Olympus FluoView 1000 confocal laser microscope (Olympus, Shinjuku, Tokyo, Japan) with lasers at the wavelengths of 405 nm, 488 nm, and 559 nm and a 40× objective (HC PL APO CS2, oil NA: 1.30). Acquisition was performed using the Olympus FV10-ASW software (v3.0; Olympus, Shinjuku, Tokyo, Japan) as z-stack images. Images were obtained in the Olympus .oib format, processed and analyzed using ImageJ software (v1.54; NIH, Bethesda, MD, USA), and displayed as a maximum intensity projection.

#### 2.8.2. Analysis of ER-Phagy in Motoneurons

The number of RFP^+^GFP^+^ or RFP^+^GFP^−^ punctae that overlapped with Lamp1^+^ vesicles were manually counted using the “cell counter” plug-in from ImageJ software (v1.54; NIH, Bethesda, MD, USA). Lamp1 was used as a marker for degradative organelles to avoid measuring false-positive RFP^+^GFP^+^ structures that might have represented vesicles traveling between the ER and Golgi.

#### 2.8.3. Analysis of IRE1α and ATF6 Localization in Early Cortical Neurons

The Golgi and nucleus were identified using the markers GM130 and DAPI, respectively. Using ImageJ software (v1.54; NIH, Bethesda, MD, USA), selection masks were generated from these marker stains. The GFP signal intensity was then quantified within the respective selections only.

### 2.9. Analysis of ER Stress Markers in Calnexin Knockout Cells

#### 2.9.1. Pharmacological Treatment with ER Stress Inducing Agents

For the analysis of ER stress upon calnexin knockout, cells were plated at 50,000 cells/well in a 12-well plate and 3 days later treated with the ER stress-inducing agents tunicamycin (final conc. 1.5 µg/mL), thapsigargin (#BML-PE180; Enzo Life Sciences, Loerrach, Germany) (final conc. 750 nM), or DTT (final conc. 5 mM) for the indicated time spans after 12 h withdrawal of growth factors.

#### 2.9.2. Protein Extraction and Analysis

At the indicated time points, cells were lysed by adding 70 µL of 2 × Laemmli buffer to the cells and heating the cell lysate to 95 °C for 5 min. Samples were homogenized using sonification and subjected to polyacrylamide gel electrophoresis. Proteins were then blotted onto PVDF membranes (#1620177; Bio-Rad, Munich, Germany), and the membranes were subsequently blocked using 5% skim milk powder (wt/vol) (#T145; Carl Roth, Karlsruhe, Germany) in TBS-T for 90 min. The membranes were washed twice with TBS-T, and primary antibodies were added overnight at 4 °C in TBS-T. The antibodies used were the following: rabbit anti-phospho-PERK (#3179, 1:1000; Cell Signaling Technologies, Frankfurt am Main, Germany), rabbit anti-phospho-eIF2α (#3597, 1:4000; Cell Signaling Technologies, Frankfurt am Main, Germany), rabbit anti-eIF2α (#5324, 1:10,000; Cell Signaling Technologies, Frankfurt am Main, Germany), mouse anti-Chop (#2895, 1:2000; Cell Signaling Technologies, Frankfurt am Main, Germany), mouse anti-Gapdh (#CB1001, 1:4000; Merck, Darmstadt, Germany), and goat anti-calnexin (#AB0041, 1:10,000; Sicgen, Cantanhede, Portugal). Then, the membrane was washed twice with TBS-T, and peroxidase (POD)-coupled secondary antibodies were added for 1 h at RT in TBS-T. The antibodies used were the following: goat anti-mouse POD (#115-035-003, 1:10,000; Jackson ImmunoResearch, West Grove, PA, USA), donkey anti-rabbit POD (#111-035-003, 1:10,000; Jackson ImmunoResearch, West Grove, PA, USA), and donkey anti-goat POD (#705-005-003, 1:10,000; Jackson ImmunoResearch, West Grove, PA, USA). Subsequently, membranes were washed four times with TBS-T and the proteins were detected using Immobilon^®^ Western HRP substrate luminol reagent and peroxide solution (#WBKLS0500; Merck, Darmstadt, Germany) and X-ray films (#4741019289; Fujifilm, Tokyo, Japan). Band intensities were quantified using ImageJ software (v1.54; NIH, Bethesda, MD, USA).

#### 2.9.3. RNA Purification and Quantification

At the indicated time points, cells were lysed, and RNA was purified using the NucleoSpin^®^ RNA Kit (#740955; Macherey-Nagel, Dueren, Germany). Subsequently, complementary DNA (cDNA) was produced with the First Strand cDNA Synthesis Kit (#K1612; Thermo Fisher Scientific, Waltham, MA, USA) using 100 ng RNA and random hexamer primers. The cDNA was then diluted 1:5 with H_2_O, and real-time quantitative PCR (RT-qPCR) was performed using Luminaris HiGreen (#K0991; Thermo Fisher Scientific, Waltham, MA, USA) and the following primers: *Xbp1*-spliced FWD, 5′-GAGTCCGCAGCAGGTG-3′; *Xbp1*-spliced REV, 5′-GTGTCAGAGTCCATGGGA-3′; *Xbp1*-total FWD, 5′-CACCTTCTTGCCTGCTGGAC-3′; *Xbp1*-total REV, 5′-GGGAGCCCTCATATCCACAGT-3′; Gapdh FWD, 5′-GCAAATTCAACGGCACA-3′; and Gapdh REV, 5′-CACCAGTAGACTCCACGAC-3′. PCR was performed using a LightCycler^®^ 96 (Roche Diagnostics, Mannheim, Germany) and LightCycler^®^ 480 Multiwell Plates 96 (#04729692001; Roche Diagnostics, Mannheim, Germany). For the quantification of the expression relative to Gapdh, the 2ΔΔCT method was used.

### 2.10. Analysis of Proteasomal and Autophagic Pathways upon ER Stress

#### 2.10.1. Pharmacological Treatment with Blockers of the Proteasome or Autophagy

For the analysis of proteasomal or autophagic pathways upon calnexin knockout, cells were plated at 350,000 cells in 6 cm culture dishes and 3 days later treated with the ER stress-inducing agent tunicamycin (final conc. 1.5 µg/mL) for 3.5 h after 12 h withdrawal of growth factors. Then, either bafilomycin A1 (#BML-CM110; Enzo Life Sciences, Loerrach, Germany) (final conc. 100 nM) or MG132 (#BML-PI102; Enzo Life Sciences, Loerrach, Germany) (final conc. 10 μM) was added for another 3.5 h.

#### 2.10.2. Protein Extraction and Analysis

After the treatment, cells were lysed with 300 µL sucrose buffer (#1.07654, 250 mM sucrose; Merck, Darmstadt, Germany), 50 mM Tris-HCl (#9090; Carl Roth, Karlsruhe, Germany), 1 mM EDTA (#CN06; Carl Roth, Karlsruhe, Germany), 0.5% Triton X-100 (vol/vol), and protease inhibitor (#5892970001; Roche Diagnostics, Mannheim, Germany), pH 7.4). Lysates were centrifuged at 13,000× *g* for 15 min at 4 °C. The supernatant (Triton X-100-soluble fraction) was collected, and the pellet was resuspended in 100 µL 1% SDS (wt/vol) (#A7249; AppliChem, Darmstadt, Germany) in PBS. The pellet was dissolved by heating to 95 °C for 5 min and sonification (Triton X-100-insoluble fraction). The samples were mixed 5:1 with 5 × Laemmli buffer and subjected to Western blot analysis (see above). The primary antibodies used were the following: mouse anti-ubiquitin (#BML-PW8805, 1:4000; Enzo Life Sciences, Lörrach, Germany), guinea pig anti-p62 (#GP62-C, 1:2000; Progen Biotechnik, Heidelberg, Germany), rabbit anti-LC3B (#NB100-2220, 1:2000; Novus Biologicals, Centennial, CO, USA), and mouse anti-Gapdh (#CB1001, 1:4000; Merck, Darmstadt, Germany). The secondary antibodies used were the following: goat anti-mouse POD (#115-035-003, 1:10,000; Jackson ImmunoResearch, West Grove, PA, USA), donkey anti-rabbit POD (#111-035-003, 1:10,000; Jackson ImmunoResearch, West Grove, PA, USA), and donkey anti-guinea pig POD (#706-005-148, 1:10,000; Jackson ImmunoResearch, West Grove, PA, USA).

## 3. Results

### 3.1. ER Stress Triggers ER-Phagy in Motoneuron Axons

**Figure 1:**
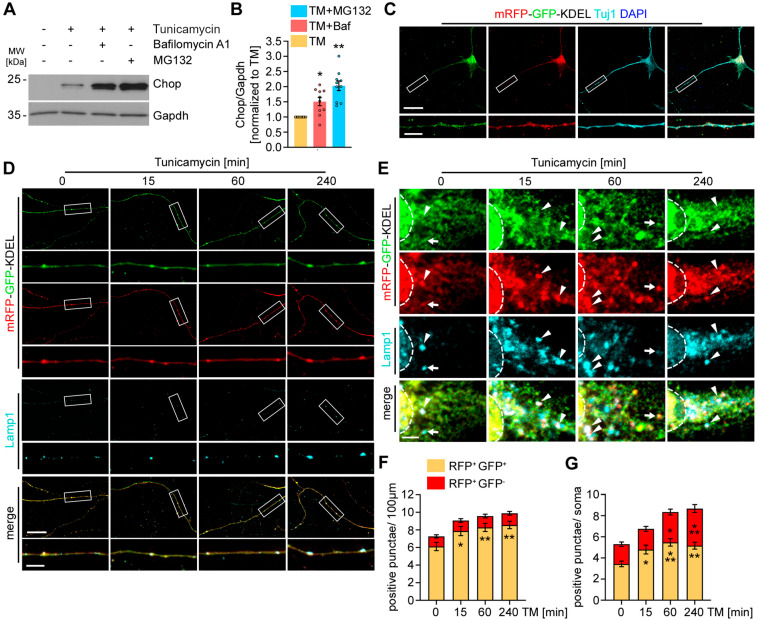
ER stress triggers local ER-phagy in the axons of primary motoneurons. (**A**) Blocking protein degradation causes enhanced UPR activation. Western blot showing the levels of Chop after tunicamycin treatment for 8 h and simultaneous treatment with bafilomycin A1 or MG132 during the last 4 h. (**B**) Quantification of the Chop intensity. The Chop levels were normalized to Gapdh and the tunicamycin condition was set to 1 in each experiment. (**C**) Expression of mRFP-GFP-KDEL in primary motoneurons showing the distribution of mRFP-GFP-KDEL to the axon and soma. Scale bar: 20 µm. Inset; Scale bar: 5 µm (**D**) Treatment with tunicamycin causes an increase in Lamp1^+^mRFP^+^GFP^+^ vesicles in the axons of primary motoneurons. Scale bar: 20 µm. Inset; Scale bar: 5 µm. (**E**) In the somata of cultured motoneurons, treatment with tunicamycin resulted in an increase in Lamp1^+^mRFP^+^GFP^+^ and Lamp1^+^mRFP^+^GFP^−^ vesicles. Arrowheads point to mRFP^+^GFP^+^ punctae. Arrows point to mRFP^+^GFP^−^ punctae. The dashed lines label the border between the cytosol and the nucleus. Scale bar: 2.5 µm (**F**,**G**) Quantification of mRFP^+^GFP^−^ and mRFP^+^GFP^+^ punctae in the axons (**F**) and somata (**G**) of primary motoneurons treated with tunicamycin for the indicated times or left untreated. Four independent experiments with at least 15 neurons analyzed. One-way ANOVA, Dunnett’s multiple comparisons test. All data in Figure 1 are shown as the mean ± SEM. * *p* < 0.05, ** *p* < 0.01, *** *p* < 0.001.

ER-phagy has been implicated as a mechanism to counteract ER stress by delivering misfolded proteins to ERLAD [28]. To examine the connection between ER-phagy and the UPR, we chemically induced ER stress by treating cultured MNs with tunicamycin. To assess the contribution of autophagy in the clearance of ER stress, we blocked lysosomal degradation using bafilomycin A1. To examine the contribution of the proteasome in ameliorating ER stress, we blocked the proteasomal turnover by treatment with MG132, interfering with ERAD. As a readout, we analyzed the expression of Chop by Western blot (Figure 1A). Blocking either lysosomal or proteasomal degradation caused a significant increase in the expression of Chop (Figure 1B), confirming that both pathways contribute to reducing the load of misfolded proteins from the ER.

Next, we asked whether ER stress was differentially regulated in subcompartments of neurons and investigated the local induction of ER-phagy in axons of cultured MNs. To monitor ER-phagy, we generated and lentivirally expressed an mRFP-GFP-KDEL tandem reporter in primary motoneurons. Upon delivery of ER fragments to lysosomes, the fluorescence of GFP is immediately quenched due to the acidic environment in the lysosome, whereas that of mRFP is stable. Therefore, ER structures delivered for lysosomal degradation only emit the mRFP signal [34].

We confirmed the localization of mRFP-GFP-KDEL to axons by co-staining with Tuj1 (Figure 1C). Under basal conditions, m RFP^+^GFP^−^ structures were hardly detectable in axons (Figure 1D). However, we frequently detected vesicular mRFP^+^GFP^+^ structures that stained positive for the late endosomal/lysosomal marker Lamp1 (Figure 1D), suggesting the delivery of ER fragments to autophagosomes, which underwent fusion with late endosomes during retrograde transport. These data are in agreement with earlier work, demonstrating the compartmentalization of autophagy in neurons [35,36]. Autophagosomes are predominantly generated in the distal part of axons, followed by fusion with late Lamp1^+^ endosomes during retrograde transport along the axons. The fusion of these vesicles with lysosomes only occurs when they have arrived in the proximal part of axons and the soma, where lysosomal degradation then occurs [35]. To explore the effect of ER stress on axonal ER-phagy, we treated primary MNs with tunicamycin for 15, 60, and 240 min and analyzed the number of mRFP^+^GFP^−^ and mRFP^+^GFP^+^ punctae, which stained positive for Lamp1 (Figure 1D,E). Upon ER stress induction, we observed an increase in Lamp1^+^mRFP^+^GFP^+^ vesicles over time in axons, while the number of Lamp1^+^mRFP^+^GFP^−^ remained unchanged in the axonal compartment (Figure 1F,G). In contrast, in the somata, ER stress induction triggered an increase in both Lamp1^+^mRFP^+^GFP^+^ and Lamp1^+^mRFP^+^GFP^−^- punctae, indicating that enhanced generation of Lamp1^+^mRFP^+^GFP^+^ vesicles in axons does not lead to any enhanced early fusion with lysosomes in axons, and that under these conditions, the fusion with lysosomes in cell bodies is enhanced.

In summary, these data suggest that ER stress induction triggers ER-phagy in axons and the soma, resulting in the delivery of ER fragments to lysosomal degradation. Based on the restricted increase in Lamp1^+^mRFP^+^GFP^−^ structures at the axon, our data also suggest that the lysosomal degradation is limited to the somata.

### 3.2. Calnexin Depletion Results in Enhanced ER-Phagy and Protein Degradation

Recent work has demonstrated an essential role of calnexin in ER-phagy, delivering proteins to the ERLAD pathway [24,25,26,27]. However, this function of calnexin has not been investigated under ER stress conditions in neurons. To analyze the ER-phagy, we isolated MNs from the control and calnexin-deficient embryos, transduced primary MNs with the mRFP-GFP-KDEL reporter, and cultured the cells for five days. ER stress was induced by treatment with tunicamycin.

**Figure 2:**
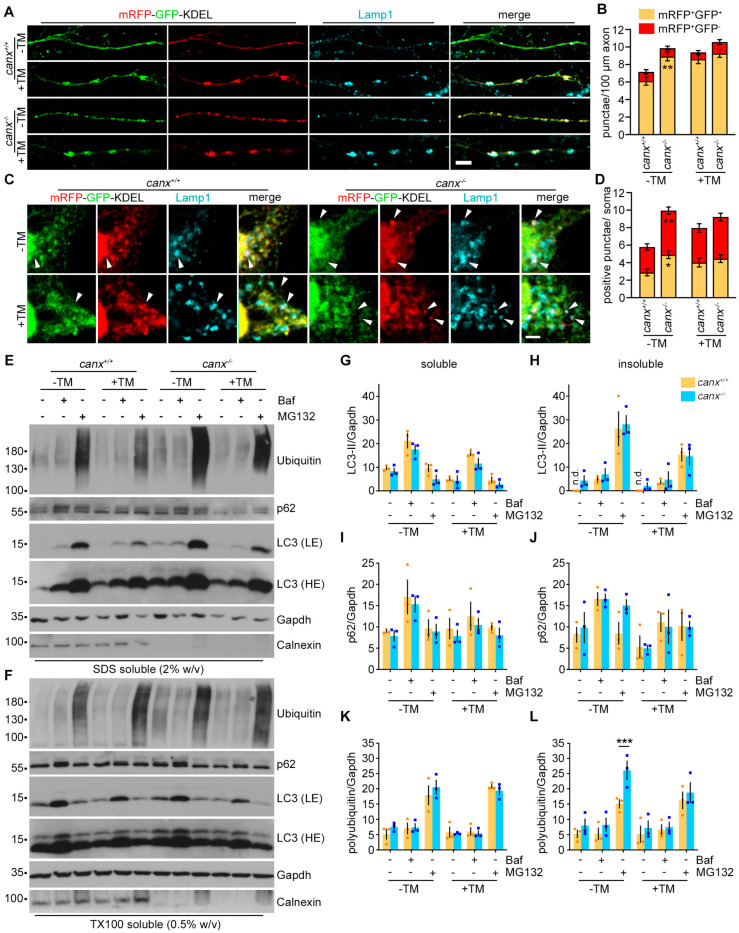
Absence of calnexin causes enhanced ER-phagy and protein degradation by default. (**A**) Calnexin depletion results in an increase in Lamp1^+^mRFP^+^GFP^+^ vesicles in the axons of primary MNs under basal conditions. Scale bar: 5 µm. mRFP-GFP-KDEL expressing MNs were cultured for 5 days and treated with tunicamycin for 1 h or left untreated. TM, Tunicamycin. (**B**) Quantification of mRFP^+^GFP^−^ and mRFP^+^GFP^+^ punctae in axons of primary MNs treated with tunicamycin for 1 h or left untreated. At least 15 neurons were analyzed per experiment. Two-way ANOVA, Tukey’s multiple comparisons test. (**C**) In the somata of cultured MNs, the loss of calnexin resulted in an increase in Lamp1^+^mRFP^+^GFP^+^ and Lamp1^+^mRFP^+^GFP^−^ vesicles under basal conditions. Arrowheads point to mRFP^+^GFP^+^ punctae. mRFP-GFP-KDEL expressing MNs were cultured for 5 days and treated with tunicamycin for 1 h or left untreated. TM, Tunicamycin. Scale bar: 2.5 µm. (**D**) Quantification of mRFP^+^GFP^−^ and mRFP^+^GFP^+^ punctae in the somata of primary MNs treated with tunicamycin for 1 h or left untreated. At least 15 neurons were analyzed per experiment. Two-way ANOVA, Tukey’s multiple comparisons test. (**E**,**F**) Western blot showing the insoluble (**E**) and soluble (**F**) protein levels of LC3, p62, and polyubiquitinated proteins. Wildtype and calnexin-deficient early cortical neurons were treated as indicated and subjected to the fractionation procedure. (**G**–**L**) Quantification of the LC3-II levels in the TX100-soluble (**G**) and SDS-soluble (**H**) fraction. Quantification of the p62 levels in the TX100-soluble (**I**) and SDS-soluble (**J**) fractions. Quantification of the polyubiquitinated protein levels in the TX100-soluble (**K**) and SDS-soluble (**L**) fractions. The protein levels were normalized to Gapdh in the soluble fraction. Three-independent experiments. *n* = 3. All data in Figure 2 are shown as the mean ± SEM. * *p* < 0.05, ** *p* < 0.01, *** *p* < 0.001.

Under basal conditions, we detected an elevated number of Lamp1^+^mRFP^+^GFP^+^ vesicles in the axons of calnexin-deficient primary MNs (Figure 2A,B). The number of Lamp1^+^mRFP^+^GFP^−^ vesicles in the MN axons was unaffected by the depletion of calnexin (Figure 2A,B). Upon treatment with tunicamycin, we observed an increase in Lamp1^+^mRFP^+^GFP^+^ vesicles in wildtype MNs, confirming the induction of ER-phagy by ER stress. However, we did not detect such an increase in calnexin-deficient MNs (Figure 2A,B). In the somata of untreated calnexin-deficient MNs, we observed a significantly elevated number of both Lamp1^+^mRFP^+^GFP^+^ and Lamp1^+^mRFP^+^GFP^−^ vesicles (Figure 2C,D). In wildtype neurons, treatment with tunicamycin caused an increase in ER-phagy, which was not detectable in calnexin-deficient cells (Figure 2C,D). In summary, these data indicate elevated ER-phagy in calnexin-deficient cells by default.

Next, we analyzed the protein turnover under basal and ER stress conditions in wildtype and calnexin-deficient early cortical neurons by Western blot (Figure 2E–L). To measure the autophagic flux, we treated the cells with bafilomycin A1, which blocks the acidification of lysosomes, leading to an enrichment of autophagosomes. To analyze the turnover by the proteasome, we treated the cells with the proteasome inhibitor MG132. Subsequently, we analyzed the Triton-X 100 soluble and insoluble protein fractions by Western blot (Figure 2E,F).

Upon treatment with tunicamycin, we observed a reduction in the autophagosomal marker LC3-II in the soluble fraction, suggesting an acceleration of the autophagic flux under ER stress conditions (Figure 2G). Indeed, exposure to bafilomycin A1 caused an enrichment of LC3-II with and without tunicamycin treatment (one-way ANOVA with Tukey’s multiple comparisons test: *canx^+/+^* −TM −Baf −MG vs. *canx^+/+^* −TM +Baf −MG: *p* = 0.0028; *canx^+/+^* +TM −Baf −MG vs. *canx^+/+^* +TM +Baf −MG: *p* = 0.0063; *canx^−/−^* −TM −Baf −MG vs. *canx^−/−^* −TM +Baf −MG: *p* = 0.0301; *canx^−/−^* +TM −Baf −MG vs. *canx^−/−^* +TM +Baf −MG: *p* = 0.1684), confirming an accelerated flux, and not reduced autophagosome formation (Figure 2G). We detected no differences in the soluble levels of LC3-II between wildtype and knockout cells. Insoluble LC3-II was absent from wildtype cells under basal and ER stress conditions. However, in calnexin-deficient cells, insoluble LC3-II was already detectable under basal conditions, suggesting an accelerated autophagosomal turnover (Figure 2H). Treatment with bafilomycin A1 caused a slight increase in insoluble LC3-II in wildtype and calnexin-deficient cells (Figure 2H).

As expected, exposure to bafilomycin A1 caused an enrichment of the soluble and insoluble p62 levels (Figure 2I,J) (one-way ANOVA with Tukey’s multiple comparisons test: soluble: *canx^+/+^* −TM −Baf −MG vs. *canx^+/+^* −TM +Baf −MG: *p* = 0.2703; *canx^−/−^* −TM −Baf −MG vs. *canx^−/−^* −TM +Baf −MG: *p* = 0.3811; insoluble: *canx^+/+^* −TM −Baf −MG vs. *canx^+/+^* −TM +Baf −MG: *p* = 0.4079; *canx^−/−^* −TM −Baf −MG vs. *canx^−/−^* −TM +Baf −MG: *p* = 0.7057). However, we did not observe any changes in the levels of p62 under ER stress conditions. We also did not detect any differences in the p62 levels between wildtype and calnexin-deficient cells (Figure 2I,J), most likely reflecting that rather ER-related cargos and not p62-dependent cytosolic cargos are sorted into autophagosomes upon ER stress.

Neither treatment with bafilomycin A1 nor tunicamycin affected the soluble polyubiquitinated protein levels. Furthermore, we did not detect any differences between wildtype and calnexin-deficient cells (Figure 2K).

In contrast to inhibiting lysosomal degradation, blocking the proteasome resulted in a marked accumulation of insoluble LC3-II (Figure 2H) (one-way ANOVA with Tukey’s multiple comparisons test: *canx^+/+^* −TM −Baf −MG vs. *canx^+/+^* −TM −Baf +MG: *p* = 0.0002; *canx^+/+^* +TM −Baf −MG vs. *canx^+/+^* +TM −Baf +MG: *p* = 0.065; *canx^−/−^* −TM −Baf −MG vs. *canx^−/−^* −TM −Baf +MG: *p* = 0.0007; *canx^−/−^* +TM −Baf −MG vs. *canx^−/−^* +TM −Baf +MG: *p* = 0.2224), without affecting the levels of soluble LC3-II (Figure 2K). Similar observations were previously made in SN56 and SH-SY5Y cells [37,38]. In contrast to LC3-II, the levels of p62 were not affected upon exposure to MG132 (Figure 2I).

As expected, inhibition of the proteasome caused a marked enrichment in the soluble and insoluble levels of polyubiquitinated proteins (Figure 2K,L) (one-way ANOVA with Tukey’s multiple comparisons test: soluble: *canx^+/+^* −TM −Baf −MG vs. *canx^+/+^* −TM −Baf +MG: *p* = 0.0005; *canx^+/+^* +TM −Baf −MG vs. *canx^+/+^* +TM −Baf +MG: *p* < 0.0001; *canx^−/−^* −TM −Baf −MG vs. *canx^−/−^* −TM −Baf +MG: *p* = 0.0004; *canx^−/−^* +TM −Baf −MG vs. *canx^−/−^* +TM −Baf +MG: *p* = 0.001; insoluble: *canx^+/+^* −TM −Baf −MG vs. *canx^+/+^* −TM −Baf +MG: *p* = 0.148; *canx^+/+^* +TM −Baf −MG vs. *canx^+/+^* +TM −Baf +MG: *p* = 0.072; *canx^−/−^* −TM −Baf −MG vs. *canx^−/−^* −TM −Baf +MG: *p* = 0.0005; *canx^−/−^* +TM −Baf −MG vs. *canx^−/−^* +TM −Baf +MG: *p* = 0.0507). Strikingly, upon inhibition of the proteasome, we observed a markedly increased accumulation of insoluble polyubiquitinated proteins in the absence of calnexin (Figure 2L). This difference was not apparent under ER stress conditions (Figure 2L), suggesting a compensatory role of the autophagy pathway.

In summary, these data indicate that calnexin deficiency causes an increased proteasomal turnover of insoluble, most likely misfolded proteins. The accumulation of insoluble LC3-II in calnexin-deficient early cortical neurons suggests an activated autophagy pathway to facilitate protein degradation by default. The results are in line with our finding of enhanced ER-phagy in calnexin-deficient MNs.

### 3.3. Calnexin Modulates the UPR

Protein misfolding causes ER stress, which triggers the UPR as an adaptive cellular response. Given the elevated protein turnover and ER-phagy in calnexin-deficient neurons, we asked whether the UPR was already active by default in calnexin-deficient neurons.

**Figure 3:**
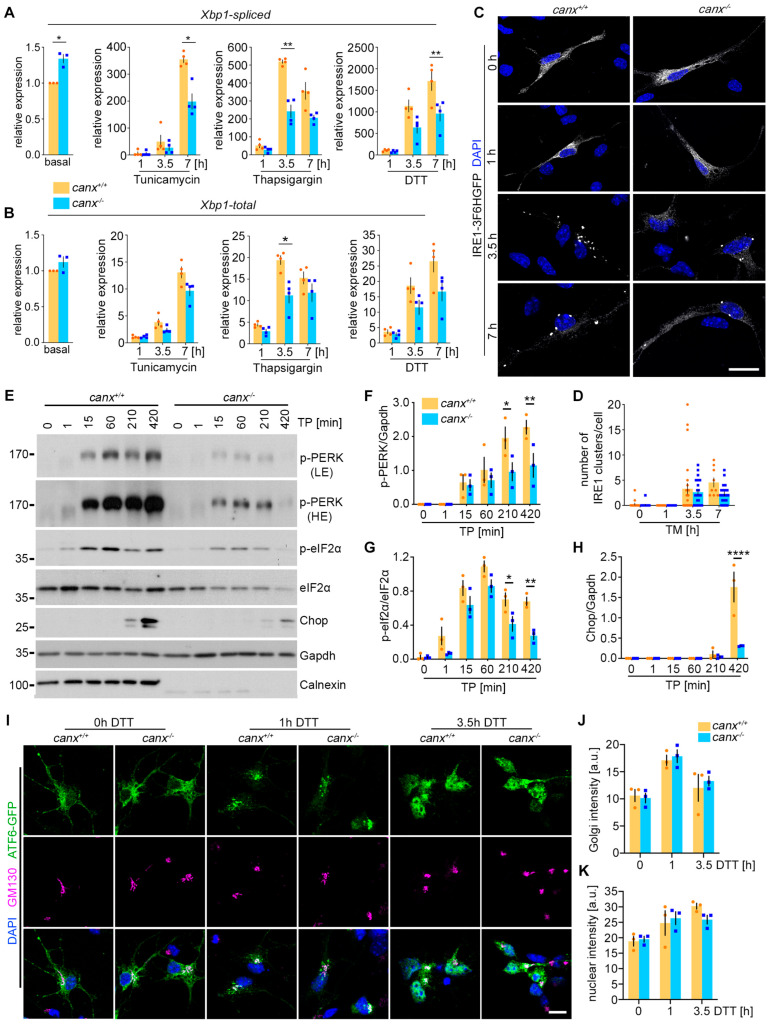
Calnexin depletion leads to a diminished activation of the PERK branch of the UPR under ER stress conditions. (**A**,**B**) Expression levels of spliced (**A**) and total *Xbp1* (**B**) in wildtype and calnexin-deficient cortical neurons analyzed by qRT-PCR. Basal expression levels were normalized to Gapdh, and *canx^+/+^* was set to 1 for each experiment. *n* = 3, One-sample *t*-test. ER stress-induced expression levels were normalized to Gapdh, and the untreated control condition was set to 1 in each experiment. Four independent experiments, *n* = 4. Two-way ANOVA, Šídák’s multiple comparisons test. (**C**) Reduced clustering of IRE1-3F6HGFP in calnexin-deficient early cortical neurons after treatment with tunicamycin. IRE1-3F6HGFP was lentivirally expressed in early cortical neurons and the cells were exposed to tunicamycin for the indicated times. Representative images are shown. Scale bar: 20 µm. (**D**) Quantification of IRE1-3F6HGFP clustering. *n* = 1, each data point represents one quantified cell. (**E**) Western blot showing an impaired activation of the PERK UPR branch in calnexin-deficient early cortical neurons upon thapsigargin treatment. The cells were exposed to thapsigargin for the indicated times, lysed, and processed for Western blotting. Western blots were probed with the indicated antibodies. (**F**–**H**) Western blot quantifications revealed a reduced phosphorylation of PERK (**F**) and eIF2α (**G**) and a reduced expression of Chop (**H**) in *canx^−/−^* cells upon ER stress induction by thapsigargin. Three independent experiments, *n* = 3. Two-way ANOVA, Šídák’s multiple comparisons test. (**I**) Deficiency in calnexin did not cause alterations in the translocation of ATF6 to the Golgi and nucleus. ATF6-EGFP was lentivirally expressed in early cortical neurons and GM130 was visualized by immunocytochemical staining. The cells were exposed to DTT for the indicated times. Scale bar: 20 µm. (**J**,**K**) Quantification of the EGFP fluorescent intensity at the Golgi (**J**) and in the nucleus (**K**). The intensity of the GFP signal overlapping with GM130 (Golgi) and DAPI (nucleus) was analyzed. Three independent experiments, *n* = 3. Two-way ANOVA, Šídák’s multiple comparisons test. All data in Figure 3 are shown as the mean ± SEM. * *p* < 0.05, ** *p* < 0.01, **** *p* < 0.0001.

To answer this question, we measured the total (*Xbp1t*) and spliced (*Xbp1s*) levels of *Xbp1* by qPCR under basal conditions, and upon chemically induced ER stress by thapsigargin, tunicamycin, and DTT, we analyzed the activation of the IRE1α branch of the UPR (Figure 3A–D). Interestingly, we detected significantly higher basal levels of *Xbp1s* upon calnexin knockout, without any significant changes in the expression of *Xbp1t* (Figure 3A,B). These data suggest a basal activation of the UPR upon depletion of calnexin.

Upon chemical UPR induction, all reagents induced a marked increase in the *Xbp1s* levels, albeit with different kinetics (Figure 3A). Whereas tunicamycin gave a slower response, the response to DTT and thapsigargin was more rapid, reflected by a faster splicing of *Xbp1*. Depletion of calnexin resulted in significantly reduced *Xbp1s* levels after 3.5 h of thapsigargin exposure and 7 h of DTT or tunicamycin exposure (Figure 3A). Chemically induced ER stress also resulted in an upregulation of *Xbp1t*, albeit less dramatic than the induction of *Xbp1s*. In calnexin-deficient cells, we also observed a significantly reduced expression of *Xbp1t* after 3.5 h of thapsigargin exposure (Figure 3B). To analyze whether the reduced expression of *Xbp1s* in calnexin-deficient cells resulted from impaired IRE1α activation, we expressed IRE1α-3F6H-GFP in wildtype and calnexin-deficient early cortical neurons and treated the cells with tunicamycin to induce ER stress. Upon activation, IRE1α oligomerized, reflected by dynamic clustering [39]. Therefore, we analyzed the clustering of IRE1α in wildtype and calnexin-deficient early cortical neurons after 1 h, 3.5 h, and 7 h of tunicamycin exposure (Figure 3C,D). In untreated cells and 1 h after the treatment with tunicamycin, IRE1α was evenly distributed in an ER-like pattern. After 3 h and 7 h of tunicamycin exposure, IRE1α appeared in a clustered pattern, which indicated its dimerization and activation. In calnexin-deficient cells, we observed a clear reduction in IRE1α clusters 3 h and 7 h after exposure to tunicamycin (Figure 3D). In summary, these results suggest that the full activation of IRE1α upon ER stress requires calnexin, leading to reduced levels of *Xbp1s* under ER stress conditions in calnexin-deficient cells.

To analyze the activation of the PERK branch of the UPR, we chemically induced ER stress with thapsigargin and measured the phosphorylation of PERK and its downstream targets eIF2α and Chop by Western blotting over a time course of 7 h. In untreated wildtype and calnexin-deficient cells, we did not detect any phosphorylation of PERK, confirming that calnexin depletion does not lead to UPR activation by default (Figure 3E). After 15 min of thapsigargin exposure, we detected phosphorylated PERK (p-PERK) in wildtype calnexin-deficient early cortical neurons, which increased over time, reaching a plateau after 3.5 h and 7 h (Figure 3F). Notably, we detected markedly reduced levels of p-PERK in calnexin-deficient cells upon exposure to thapsigargin (Figure 3E,F). Correlated with these results, we also observed a reduced phosphorylation of eIF2α and a reduced expression of Chop in calnexin-deficient cells under ER stress conditions (Figure 3G,H). In summary, these data suggest that calnexin modulates the activation of PERK upon ER stress induction. Next, we wondered whether the activation of ATF6 similarly depended on calnexin.

In contrast to the dimerization and autophosphorylation of IRE1α and PERK under ER stress conditions, ATF6 is activated by a different mechanism. Upon ER stress, ATF6 moves from the ER to the Golgi, where it is processed into ATF6p50, which translocates to the nucleus [15]. To analyze the activation of ATF6, we lentivirally expressed ATF6-GFP in wildtype and calnexin-deficient early cortical neurons and measured the localization of ATF6-GFP upon exposure to DTT for 1 h and 3.5 h (Figure 3I–K). Due to the lack of Tunicamycin in triggering the activation of ATF6 as previously described [15], we used DTT for these experiments. Under control conditions, ATF6-GFP showed an ER-like pattern and was mostly absent from the nuclei (Figure 3I). After 1 h of DTT treatment, we detected a marked accumulation of the GFP signal at the Golgi apparatus, as shown by co-staining with GM130 (Figure 3I). From 1 h to 3.5 h, the GFP signal shifted from the Golgi to the nucleus, confirming the transit from the ER to the Golgi, followed by proteolytic processing and translocation to the nucleus [15]. Interestingly, we did not detect any significant differences in the activation of ATF6 between wildtype and calnexin-deficient cells (Figure 3J,K).

In summary, this data set suggests that in contrast to PERK and IRE1α, the function of calnexin seems to not be critical in ATF6 activation under ER stress conditions.

## 4. Discussion

In summary, our data demonstrate that calnexin plays an essential role in mediating ER-phagy and the UPR in neurons. ER stress is a major pathophysiological pathway linked to motoneuron dysfunction and loss in amyotrophic lateral sclerosis (ALS) [6]. Considering that axonal defects and presynaptic dysfunction precede motoneuron loss [40], it is of particular interest to understand the local axonal mechanism to counteract ER stress and maintain ER homeostasis. Interestingly, ER proteins are among the most abundant cargo in purified brain autophagosomes [41], suggesting a critical role for ER-phagy in maintaining ER homeostasis.

Our data demonstrate that ER stress triggers the delivery of axonal ER fragments to lysosomal degradation, pointing towards an essential role for ER-phagy in the turnover of the ER under stress conditions. In agreement with the compartmentalized autophagy in neurons [35], we mostly detected ER fragments in Lamp1+ vesicles, which had not been acidified. These results suggest that ER fragments are delivered to autophagosomes, which rapidly fuse with late endosomes [35], followed by retrograde transport and lysosomal turnover at the somata. However, to confirm the involvement of macro-ER-phagy, which involves the lipidation of LC3, further experiments are required.

Our results showed that the absence of calnexin leads to an increased ER-phagy and proteasomal turnover, suggesting that the depletion of calnexin already results in elevated protein misfolding by default. We detected considerable amounts of LC3-II in the SDS-soluble fraction only upon blocking proteasomal degradation. This observation has been made previously in SN56 and SH-SY5Y cells [37,38]. It is known that the autophagosomal system can compensate for impaired proteasome function and that this system is activated upon proteasome inhibition [42,43]. As a result, the increase in SDS-soluble LC3-II might reflect aggregated autophagosomal components which result from an overload of the lysosomal system caused by proteasomal inhibition.

The basal UPR activation via the IRE1α branch was higher in calnexin knockout cells as shown by the increased splicing of *Xbp1*. Similar observations were previously made for calnexin-deficient fibroblasts, where basal *Xbp1s* levels were increased and the capability to activate the UPR upon thapsigargin treatment was slightly reduced [44]. However, we were not able to detect an increased basal UPR activation of the PERK or ATF6 branch. This might be due to the limitations of our approaches via Western blot and Immunostaining, providing too little sensitivity for subtle changes. This is in contrast to *Xbp1s* qPCR, which is a highly sensitive method for detecting already minor changes in mRNA levels. The increased UPR activation in the IRE1α branch presumably leads to an activation of both ER-associated degradation pathways, ERLAD and ERAD. Consequently, calnexin-deficient cells might not be as severely affected by ER stress induction since the misfolded protein load is lower due to the higher turnover and perhaps also due to a reduced protein translation. As a result, the UPR activation upon ER stress is not as pronounced as in wildtype cells.

Alternatively, calnexin depletion might directly affect the activation of PERK and IRE1α. Previously, other chaperones have been identified as direct UPR modulators [45,46]. The ER resident protein disulfide isomerase A6 (PDIA6) limits the duration of IRE1α activity by directly binding to the luminal domain of IRE1α. PDIA6-deficient cells show a more pronounced response to ER stress with prolonged autophosphorylation of IRE1α [45]. The ER resident Heat Shock Protein 47 (Hsp47) also directly binds to the ER luminal domain of IRE1α to modulate its activity. However, in contrast to PDIA6, Hsp47 depletion causes a reduced IRE1α activation, demonstrating that Hsp47 facilitates the oligomerization of IRE1α. Indeed, the interaction of Hsp47 with IRE1α leads to the displacement of BiP from the complex to facilitate the oligomerization of IRE1α [46]. In a similar manner, calnexin might regulate the activation of PERK and IRE1α. The direct interaction of calnexin with the luminal or cytosolic domain of sensors might act as a mechanism, which is required to facilitate sensor oligomerization by displacing BiP from the complex with PERK and IRE1α. A specific interaction of calnexin with PERK and IRE1α might also explain why the activation of Atf6 was not affected upon calnexin depletion.

In summary, our study highlights the central role of calnexin in orchestrating ER-phagy and the UPR to maintain protein homeostasis within the ER.

## Data Availability

The original contributions presented in the study are included in the article, further inquiries can be directed to the corresponding authors.

## References

[1] Deng C., Moradi M., Reinhard S., Ji C., Jablonka S., Hennlein L., Lüningschrör P., Doose S., Sauer M., Sendtner M. (2021). Dynamic remodeling of ribosomes and endoplasmic reticulum in axon terminals of motoneurons. J. Cell. Sci..

[2] Kuijpers M., Kochlamazashvili G., Stumpf A., Puchkov D., Swaminathan A., Lucht M.T., Krause E., Maritzen T., Schmitz D., Haucke V. (2021). Neuronal Autophagy Regulates Presynaptic Neurotransmission by Controlling the Axonal Endoplasmic Reticulum. Neuron.

[3] Deng C., Reinhard S., Hennlein L., Eilts J., Sachs S., Doose S., Jablonka S., Sauer M., Moradi M., Sendtner M. (2022). Impaired dynamic interaction of axonal endoplasmic reticulum and ribosomes contributes to defective stimulus-response in spinal muscular atrophy. Transl. Neurodegener..

[4] Farias G.G., Fréal A., Tortosa E., Stucchi R., Pan X., Portegies S., Will L., Altelaar M., Hoogenraad C.C. (2019). Feedback-Driven Mechanisms between Microtubules and the Endoplasmic Reticulum Instruct Neuronal Polarity. Neuron.

[5] Wu Y., Whiteus C., Xu C.S., Hayworth K.J., Weinberg R.J., Hess H.F., De Camilli P. (2017). Contacts between the endoplasmic reticulum and other membranes in neurons. Proc. Natl. Acad. Sci. USA.

[6] Hetz C., Saxena S. (2017). ER stress and the unfolded protein response in neurodegeneration. Nat. Rev. Neurol..

[7] Hetz C., Zhang K., Kaufman R.J. (2020). Mechanisms, regulation and functions of the unfolded protein response. Nat. Rev. Mol. Cell Biol..

[8] Bertolotti A., Zhang Y., Hendershot L.M., Harding H.P., Ron D. (2000). Dynamic interaction of BiP and ER stress transducers in the unfolded-protein response. Nat. Cell Biol..

[9] Shamu C.E., Walter P. (1996). Oligomerization and phosphorylation of the Ire1p kinase during intracellular signaling from the endoplasmic reticulum to the nucleus. EMBO J..

[10] Harding H.P., Zhang Y., Bertolotti A., Zeng H., Ron D. (2000). Perk is essential for translational regulation and cell survival during the unfolded protein response. Mol. Cell.

[11] Harding H.P., Zhang Y., Ron D. (1999). Protein translation and folding are coupled by an endoplasmic-reticulum-resident kinase. Nature.

[12] Calfon M., Zeng H., Urano F., Till J.H., Hubbard S.R., Harding H.P., Clark S.G., Ron D. (2002). IRE1 couples endoplasmic reticulum load to secretory capacity by processing the XBP-1 mRNA. Nature.

[13] Sidrauski C., Walter P. (1997). The transmembrane kinase Ire1p is a site-specific endonuclease that initiates mRNA splicing in the unfolded protein response. Cell.

[14] Lee A.H., Iwakoshi N.N., Glimcher L.H. (2003). XBP-1 regulates a subset of endoplasmic reticulum resident chaperone genes in the unfolded protein response. Mol. Cell Biol..

[15] Chen X., Shen J., Prywes R. (2002). The luminal domain of ATF6 senses endoplasmic reticulum (ER) stress and causes translocation of ATF6 from the ER to the Golgi. J. Biol. Chem..

[16] Ye J., Rawson R.B., Komuro R., Chen X., Davé U.P., Prywes R., Brown M.S., Goldstein J.L. (2000). ER stress induces cleavage of membrane-bound ATF6 by the same proteases that process SREBPs. Mol. Cell.

[17] Yoshida H., Haze K., Yanagi H., Yura T., Mori K. (1998). Identification of the cis-acting endoplasmic reticulum stress response element responsible for transcriptional induction of mammalian glucose-regulated proteins. Involvement of basic leucine zipper transcription factors. J. Biol. Chem..

[18] Chevet E., Smirle J., Cameron P.H., Thomas D.Y., Bergeron J.J. (2010). Calnexin phosphorylation: Linking cytoplasmic signalling to endoplasmic reticulum lumenal functions. Semin. Cell Dev. Biol..

[19] Wada I., Imai S.-I., Kai M., Sakane F., Kanoh H. (1995). Chaperone function of calreticulin when expressed in the endoplasmic reticulum as the membrane-anchored and soluble forms. J. Biol. Chem..

[20] Wada I., Rindress D., Cameron P., Ou W., Doherty J., Louvard D., Bell A., Dignard D., Thomas D., Bergeron J. (1991). SSR alpha and associated calnexin are major calcium binding proteins of the endoplasmic reticulum membrane. J. Biol. Chem..

[21] Tannous A., Pisoni G.B., Hebert D.N., Molinari M. (2015). N-linked sugar-regulated protein folding and quality control in the ER. Semin. Cell Dev. Biol..

[22] Hammond C., Braakman I., Helenius A. (1994). Role of N-linked oligosaccharide recognition, glucose trimming, and calnexin in glycoprotein folding and quality control. Proc. Natl. Acad. Sci. USA.

[23] Paskevicius T., Farraj R.A., Michalak M., Agellon L.B. (2023). Calnexin, More Than Just a Molecular Chaperone. Cells.

[24] Luningschror P., Andreska T., Veh A., Wolf D., Giridhar N.J., Moradi M., Denzel A., Sendtner M. (2023). Calnexin controls TrkB cell surface transport and ER-phagy in mouse cerebral cortex development. Dev. Cell.

[25] Fregno I., Fasana E., Solda T., Galli C., Molinari M. (2021). N-glycan processing selects ERAD-resistant misfolded proteins for ER-to-lysosome-associated degradation. EMBO J..

[26] Forrester A., De Leonibus C., Grumati P., Fasana E., Piemontese M., Staiano L., Fregno I., Raimondi A., Marazza A., Bruno G. (2019). A selective ER-phagy exerts procollagen quality control via a Calnexin-FAM134B complex. EMBO J..

[27] Fregno I., Fasana E., Bergmann T.J., Raimondi A., Loi M., Solda T., Galli C., D’Antuono R., Morone D., Danieli A. (2018). ER-to-lysosome-associated degradation of proteasome-resistant ATZ polymers occurs via receptor-mediated vesicular transport. EMBO J..

[28] Molinari M. (2021). ER-phagy responses in yeast, plants, and mammalian cells and their crosstalk with UPR and ERAD. Dev. Cell.

[29] Tsukita S., Ishikawa H. (1976). Three-dimensional distribution of smooth endoplasmic reticulum in myelinated axons. J. Electron. Microsc..

[30] Denzel A., Molinari M., Trigueros C., Martin J.E., Velmurgan S., Brown S., Stamp G., Owen M.J. (2002). Early postnatal death and motor disorders in mice congenitally deficient in calnexin expression. Mol. Cell Biol..

[31] Luningschror P., Werner G., Stroobants S., Kakuta S., Dombert B., Sinske D., Wanner R., Lüllmann-Rauch R., Wefers B., Wurst W. (2020). The FTLD Risk Factor TMEM106B Regulates the Transport of Lysosomes at the Axon Initial Segment of Motoneurons. Cell Rep..

[32] Samtleben S., Wachter B., Blum R. (2015). Store-operated calcium entry compensates fast ER calcium loss in resting hippocampal neurons. Cell Calcium.

[33] De Lorenzo F., Luningschror P., Nam J., Beckett L., Pilotto F., Galli E., Lindholm P., Rudt von Collenberg C., Mungwa S.T., Jablonka S. (2023). CDNF rescues motor neurons in models of amyotrophic lateral sclerosis by targeting endoplasmic reticulum stress. Brain.

[34] Chino H., Mizushima N. (2020). ER-Phagy: Quality Control and Turnover of Endoplasmic Reticulum. Trends Cell Biol..

[35] Maday S., Wallace K.E., Holzbaur E.L. (2012). Autophagosomes initiate distally and mature during transport toward the cell soma in primary neurons. J. Cell Biol..

[36] Luningschror P., Binotti B., Dombert B., Heimann P., Perez-Lara A., Slotta C., Thau-Habermann N., von Collenberg C.R., Karl F., Damme M. (2017). Plekhg5-regulated autophagy of synaptic vesicles reveals a pathogenic mechanism in motoneuron disease. Nat. Commun..

[37] Gal J., Bang Y., Choi H.J. (2012). SIRT2 interferes with autophagy-mediated degradation of protein aggregates in neuronal cells under proteasome inhibition. Neurochem. Int..

[38] Bang Y., Kim K.-S., Seol W., Choi H.J. (2016). LRRK2 interferes with aggresome formation for autophagic clearance. Mol. Cell. Neurosci..

[39] Li H., Korennykh A.V., Behrman S.L., Walter P. (2010). Mammalian endoplasmic reticulum stress sensor IRE1 signals by dynamic clustering. Proc. Natl. Acad. Sci. USA.

[40] Murray L.M., Talbot K., Gillingwater T.H. (2010). Review: Neuromuscular synaptic vulnerability in motor neurone disease: Amyotrophic lateral sclerosis and spinal muscular atrophy. Neuropathol. Appl. Neurobiol..

[41] Kallergi E., Sankar D.S., Matera A., Kolaxi A., Paolicelli R.C., Dengjel J., Nikoletopoulou V. (2023). Profiling of purified autophagic vesicle degradome in the maturing and aging brain. Neuron.

[42] Wang D., Xu Q., Yuan Q., Jia M., Niu H., Liu X., Zhang J., Young C.Y., Yuan H. (2019). Proteasome inhibition boosts autophagic degradation of ubiquitinated-AGR2 and enhances the antitumor efficiency of bevacizumab. Oncogene.

[43] Li C., Wang X., Li X., Qiu K., Jiao F., Liu Y., Kong Q., Liu Y., Wu Y. (2019). Proteasome Inhibition Activates Autophagy-Lysosome Pathway Associated With TFEB Dephosphorylation and Nuclear Translocation. Front. Cell Dev. Biol..

[44] Coe H., Bedard K., Groenendyk J., Jung J., Michalak M. (2008). Endoplasmic reticulum stress in the absence of calnexin. Cell Stress Chaperones.

[45] Eletto D., Dersh D., Gidalevitz T., Argon Y. (2014). Protein disulfide isomerase A6 controls the decay of IRE1alpha signaling via disulfide-dependent association. Mol. Cell.

[46] Sepulveda D., Rojas-Rivera D., Rodriguez D.A., Groenendyk J., Köhler A., Lebeaupin C., Ito S., Urra H., Carreras-Sureda A., Hazari Y. (2018). Interactome Screening Identifies the ER Luminal Chaperone Hsp47 as a Regulator of the Unfolded Protein Response Transducer IRE1alpha. Mol. Cell.

